# Pediatric Mesenchymal Stem Cells Exhibit Immunomodulatory Properties Toward Allogeneic T and B Cells Under Inflammatory Conditions

**DOI:** 10.3389/fbioe.2019.00142

**Published:** 2019-06-12

**Authors:** Virginia Palomares Cabeza, Martin Johannes Hoogduijn, Rens Kraaijeveld, Marcella Franquesa, Janneke Witte-Bouma, Eppo B. Wolvius, Eric Farrell, Pieter A. J. Brama

**Affiliations:** ^1^Department of Oral and Maxillofacial Surgery, Erasmus University Medical Center, Rotterdam, Netherlands; ^2^Nephrology and Transplantation, Department of Internal Medicine, Erasmus University Medical Center, Rotterdam, Netherlands; ^3^School of Veterinary Medicine, University College Dublin, Dublin, Ireland; ^4^REMAR Group and Nephrology Service, Germans Trias i Pujol Health Science Institute and University Hospital, Badalona, Spain

**Keywords:** mesenchymal stem cell, immunomodulation, allogeneic, T cell, B cell, inflammatory microenvironment

## Abstract

Mesenchymal stem cells from pediatric patients (pMSCs) are an attractive cell source in regenerative medicine, due to their higher proliferation rates and better differentiation abilities compared to adult MSCs (aMSCs). We have previously characterized the immunomodulatory abilities of pMSCs on T cells under co-culture. It has also been reported that aMSCs can inhibit B cell proliferation and maturation under inflammatory conditions. In this study, we therefore aimed to clarify the immunomodulatory effect of pMSCs toward T and B cells in an inflammatory microenvironment. Bone marrow derived pMSCs were primed to simulate inflammatory conditions by exposure with 50 ng/mL of IFN-γ for 3 days. To analyze the interaction between pMSCs and T cells, CD3/CD28 stimulated peripheral blood mononuclear cells (PBMCs) were co-cultured with primed or unprimed pMSCs. To investigate B cell responses, quiescent B cells obtained from spleens by CD43 negative selection were stimulated with anti-IgM, anti-CD40, IL-2, and co-cultured with either IFN-γ primed or unprimed pMSC. pMSC phenotype, B and T cell proliferation, and B cell functionality were analyzed. Gene expression of indoleamine 2,3-dioxygenease (IDO), as well as the expression of HLA-ABC, HLA-DR and the co-stimulatory molecules CD80 and CD86 was upregulated on pMSCs upon IFN-γ priming. IFN-γ did not alter the immunomodulatory abilities of pMSCs upon CD4^+^ nor CD8^+^ stimulated T cells compared to unprimed pMSCs. IFN-γ primed pMSCs but not unprimed pMSCs strongly inhibited naïve (CD19^+^CD27^−^), memory (CD19^+^CD27^+^), and total B cell proliferation. Antibody-producing plasmablast (CD19^+^CD27^high^CD38^high^) formation and IgG production were also significantly inhibited by IFN-γ primed pMSCs compared to unprimed pMSCs. Collectively, these results show that pMSCs have immunomodulatory effects upon the adaptive immune response which can be potentiated by inflammatory stimuli. This knowledge is useful in regenerative medicine and allogeneic transplantation applications toward tailoring pMSCs function to best modulate the immune response for a successful implant engraftment and avoidance of a strong immune reaction.

## Introduction

Mesenchymal stem cells (MSCs) are a source of self-renewing multipotent stem cells that are capable of differentiating along adipogenic, osteogenic, and chondrogenic lineages (Chamberlain et al., 2007; Bianco et al., 2008). MSCs are also known to exert potent immunomodulatory effects upon a wide range of immune cells, both of the innate and the adaptive immune system (Gao et al., 2016). This effect seems to be dependent upon the inflammatory conditions of the micro-environment in which these MSCs are found (Krampera et al., 2006; Cuerquis et al., 2014). Particularly for the adaptive immune response, pro-inflammatory cytokines, such as IFN-γ, seem to potentiate the anti-proliferative effect of MSC on T cells by inducing an increase in the activity of the enzyme indoleamine 2,3-dioxygenease (IDO) (Meisel et al., 2004). Regarding their interaction with B cells, MSCs have been described to suppress B cell proliferation through soluble factors (Corcione et al., 2006) and abrogate plasmablast formation independently of T cells (Franquesa et al., 2015). It has also been shown that upon IFN-γ stimulation, MSCs significantly inhibit B cell proliferation and maturation by upregulating IDO expression (Luk et al., 2017).

Due to their multipotent differentiation and immune modulation properties, MSCs have been successfully investigated for their use in several diseases such as ischemia (Cortez-Toledo et al., 2018), autoimmune diseases (Gerdoni et al., 2007; González et al., 2009), as well as in solid organ transplantation (Benseler et al., 2014). Other applications of MSCs include their potential use in tissue engineering and regenerative medicine (Barry and Murphy, 2013; Shao et al., 2015). In particular in this field, bone marrow derived MSCs (BM-MSCs) have been successfully differentiated toward a chondrogenic phenotype and used for *in vivo* bone formation following the process of endochondral ossification (Farrell et al., 2011; van der Stok et al., 2014). Nevertheless, the high variability between BM-MSC donors as a result of age and disease status has been shown to have an increasing importance by negatively influencing their bone formation potential in the case of elderly donors (Stolzing, 2006; Ganguly et al., 2017). Hence, a source of BM-MSCs with less age related variations are potentially more promising candidates for these applications (Stolzing, 2006). Pediatric BM-MSCs (pMSCs) obtained from iliac crest bone chips from individuals between 7 and 13 years old have increased differentiation and proliferation capacities compared to adult BM-MSCs (aMSCs) (Knuth et al., 2018). pMSCs have been described to maintain an immunophenotype identical to aMSCs and are significantly less senescent (Knuth et al., 2018).

In the context of an allogeneic transplantation, the adaptive immune response plays an important role in determining the outcome of the engraftment of the allograft (Cozzi et al., 2017). Naïve and memory CD4^+^ and CD8^+^ alloreactive T cells mediate rejection and graft-vs.-host disease processes (Cozzi et al., 2017; DeWolf and Sykes, 2017). The cross-talk between B and T cells is critical in these immune responses, since B cells are known to be the mediators of humoral rejection by producing donor-specific human leukocyte antigen (HLA) antibodies upon activation by T cells (Larsen et al., 2006).

We have previously shown that pMSCs can exert an immunomodulatory effect on T cells by reducing their proliferation rates in an *in vitro* co-culture model (Knuth et al., 2018). Since in an allogeneic transplantation setting pMSCs might be subjected to an inflammatory microenvironment their immune properties might also be altered, affecting their success for clinical uses. Hence, to characterize how the inflammatory microenvironment can affect their immune status, in this study we investigated the effect of IFN-γ priming of a novel source of pMSCs on their immunomodulatory functionality toward B and T cells.

## Methods

### Isolation and Culture of Human Pediatric Bone Marrow Derived MSCs (pMSCs)

pMSCs were isolated from leftover iliac crest bone chips of pediatric patients undergoing alveolar bone graft surgery. Written consent was not required according to institutional guidelines for the use of waste surgical material but an opt out was available. This was approved by the Erasmus Medical Ethical Committee (MEC-2014-16). The age of the patients ranged between 9 and 13 years old Detailed information about age and sex of the donors can be found in Table 1.

**Table 1:** Details of age and sex of the pMSC donors used in the study.

**Donor**	**Age (years)**	**Sex**
Donor 1	12	Male
Donor 2	12	Female
Donor 3	10	Female
Donor 4	10	Male
Donor 5	Between 9 and 13	Male
Donor 6	9	Male

Briefly, pMSCs were obtained by washing the iliac crest chips twice with 10 mL of αMEM expansion medium supplemented with 10% heat inactivated fetal bovine serum, 1.5 μg/mL Amphotericin B, 25 μg/mL L-ascorbic acid 2-phosphate, 50 μg/mL gentamycin (all from Invitrogen) and 1 ng/mL fibroblast growth factor-2 (BioRad). The medium from the two washes containing the pMSCs was then plated in T75 flasks which were washed twice 24 h after with phosphate-buffered saline (PBS) supplemented with 2% v/v heat inactivated fetal bovine serum to remove non-adherent dead cells. Viable pMSCs were at all times cultured at 37°C and 5% carbon dioxide (CO_2_) in a humidified atmosphere. Expansion medium was replaced at least twice a week and the cells were firstly passaged when several visible colonies were detected using 0.05% trypsin-EDTA (Invitrogen). Upon the second passage, cells were always trypsinized at 70–80% of confluency. The cells showed an MSC characteristic morphology and were used between passages three and five for all experiments. Their phenotypic characteristics were previously extensively described by our group (Knuth et al., 2018).

### Isolation of PBMCs From Peripheral Blood

Peripheral blood from healthy male donors was obtained from Sanquin Bloedvoorziening (Rotterdam, the Netherlands). Samples were centrifuged at 388 g for 7 min to remove the top layer of plasma and washed at a 1:2 dilution in wash medium (RPMI-1640 supplemented with 1.5 μg/mL Amphotericin B and 50 μg/mL gentamycin). The remaining cell suspension was then transferred to Ficoll-Paque PLUS (density 1.077 g/mL; GE Healthcare) containing tubes and centrifuged at 690 g for 20 min with the brake turned off. The plasma was removed and the layers above the filter were then washed with wash medium up to a total volume of 50 mL. Samples were then washed three times in wash medium as in the previous step, and then finally cells were counted and resuspended in human serum conditioned medium (PBMC medium) composed of RPMI-1640 medium with 1% v/v GlutaMAX, 1.5 μg/mL Amphotericin B, 50 μg/mL gentamycin and 10% v/v heat inactivated human serum (Sigma-Aldrich). Cells were then resuspended in PBMC medium supplemented with a 10% of dimethylsulphoxide in appropriate numbers for optimal conservation and stored in liquid nitrogen until used for the experiments.

### IFN-γ Pre-stimulation of pMSCs

Based on previous optimization experiments, pMSCs were pre-treated for 3 days using IFN-γ (50 ng/mL, Peprotech) prior to co-cultures (Luk et al., 2017). Twenty four hours before the co-culture day, cells were detached with 0.05% w/v trypsin-EDTA, washed with PBS and seeded in a 96 well plate at a density of 0.2 × 10^6^ cells per well in either 100 μL of Iscove's Modified Dulbecco's Medium (IMDM, Lonza) supplemented with a 10% v/v heat inactivated FBS (for B cell co-cultures), or in 100 μL of PBMC medium (for PBMC co-cultures).

### Quantitative Real-Time Reverse Transcription Polymerase Chain Reaction (qRT-PCR)

After 3 days of IFN-γ stimulation, 0.3 × 10^6^ pMSCs were placed in 300 μL of RLT buffer and snap frozen. RNA was isolated using a RNeasy micro kit (QIAGEN), and complementary cDNA was synthetized using a first strand cDNA kit (RevertAid cDNA kit, Thermo Scientific). qRT-PCR was performed using a 2x TaqMan Universal PCR master mix (Applied Biosystems), according to manufacturer's instructions and assay on demand primers (Thermoscientific) for IDO (Hs 00158027.m1) and for GAPDH (forward: 5′-ATGGGGAAGGTGAAGGTCG-3′, reverse: 5′-TAAAAGCAGCCCTGGTGACC-3′, probe (FAM-TAMRA): 5′ CGCCCAATACGACCAAATCCGTTGAC-3′). TAQ DNA polymerase (Hot Start) was activated for 10 min at 95°C, then DNA was amplified following 40 cycles of 15 s at 95°C, and 1 min at 60°C. TAQman was analyzed on a CFX-96 thermal cycler (BioRad). Results are expressed as relative copy number of PCR products in respect to the housekeeper GAPDH.

### pMSC Phenotyping by Flow Cytometry

pMSCs were immunophenotypically analyzed with and without IFN-γ pre-stimulation by assessing the expression of surface markers: HLA-ABC FITC, HLA-DR PerCP (clone G46.6), CD80 PE-Cy7 (clone L307.4), CD86 PE (clone 2331 FUN-1), all from BD Biosciences, San Jose, CA, USA by Flow Cytometry (FACS Jazz, BD Biosciences, San Jose, CA, USA). A total of *N* = 3 different pMSC donors in triplicates were analyzed.

### T Cell Proliferation Analysis

Isolated PBMCs were thawed in 10 mL of pre-warmed PBMC medium and centrifuged at 248 g for 8 min. Cells were counted and in order to track proliferation, they were resuspended to a concentration of 10^7^ cells/mL, and 20 μL of carboxyfluorescein succinimidyl ester (CFSE, 5 μM) were added per 0.980 μL of cell suspension for 7 min at 37°C. After that time, cell suspensions were topped up to a 10 mL volume of cold PBMC medium, and centrifuged 10 min at 690 g. T cell proliferation was stimulated using antibodies against CD3 and CD28 (1 mg/mL, 1 μL each per 10^6^ cells, BD Biosciences) and a Goat linker antibody (0.5 mg/mL, 2 μL per 10^6^ cells, BD Biosciences). Stimulated PBMCs were then added at 1:2.5, 1:5, 1:10 and 1:20 ratios to previously seeded pMSCs and co-cultured for 5 days.

PBMCs were removed by careful aspiration from the wells and washed with FACSflow. Cells were resuspended in 100 μL of FACSflow containing antibodies and fixed overnight in 4.6% paraformaldehyde. Prior to the analysis, samples were washed and resuspended in 100 μL of FACSflow. To identify T cells and specific subsets, antibodies against CD3 PerCP (clone SK7), CD4 APC (SK3) and CD8 PE-Cy7 (SK1) were used (all from BD Biosciences, San Jose, CA, USA). T cell proliferation was tracked by flow cytometry using FACS Jazz. Samples were analyzed using the software FlowJo V10.07 (BD Biosciences). *N* = 3 different pMSC donors with *N* = 3 different PBMC donors in triplicates were analyzed.

### Isolation of B Cells From Spleens

Human splenocytes were obtained from the spleens of deceased kidney donors [The Netherlands Law of organ donation (Wet op Orgaandonatie, WOD), article 13]. Ficoll-Paque (Amersham Pharmacia Biotech, Uppsala, Sweden) density gradient separation was performed on spleens segments that were previously mechanically disrupted and filtered with a 70 μM cell strainer. Mononuclear cells were stored at −150°C until the date of use. On the co-culture day, quiescent B cells were isolated from thawn splenocytes by using anti-CD43 magnetic beads (Miltenyi Biotec GmbH, Bergisch Gladbach, Germany). CD19^+^ purity was determined on the CD43^−^ fraction by flow cytometry (FACS Canto II, BD Biosciences, San Jose, CA, USA) and cells suspensions with a >97% purity were used.

### B Cell Proliferation and Subset Analysis

Purified CD43^−^ quiescent B cells were labeled with CFSE (Molecular Probes Invitrogen, Karlsruhe, Germany) as described previously for PBMC proliferation for 7 min at 37°C. After labeling, B cells were resuspended in IMDM with 10% v/v heat inactivated FBS supplemented with a cocktail to mimic T cell activation, composed of 1,000 UI/mL IL-2 (Proleukin, Novartis, Prometheus laboratories Inc., San Diego, CA, USA), 10 μg/mL Goat anti-human IgM (Jackson Immunoresearch, Cambridgeshire, UK), and 5 μg/mL soluble recombinant human CD40L (Biolegend, San Diego, CA, USA). One hundred microliter per well of the previously described cell suspension were added to the previously seeded pMSCs at a 1:5 MSC:B cell ratio and co-cultured for 7 days.

B cell proliferation was characterized by flow cytometry (FACS Canto II) and analyzed with FlowJo V10.07 (BD Biosciences). Samples were collected by careful aspiration and centrifuged for 5 min at 690 g, and supernatants were stored at −80°C for IgG quantification. Cells were stained using the following flow cytometry antibodies: CD19-BV512 (clone HIB19), CD27 PE-Cy7 (clone 0323), CD38-PE (clone HB7), Viaprobe (BD Biosciences, San Jose, CA, USA). A total of *N* = 3 different B cell donors co-cultured with one pMSC donor in duplicates or triplicates were analyzed.

### IgG ELISA

Human IgG present in the supernatants was quantified by using a Human total IgG Ready SET Go ELISA kit (Thermofisher) following the manufacturer's instructions. Briefly, ELISA plates were coated overnight in coating buffer. The next day, samples were thawed in ice and diluted 1:2 in Assay buffer. Plates were washed twice with 400 μL per well of Wash Buffer, and blocked with 250 μL of Blocking buffer. One hundred microliter of either 1:2 diluted samples or standard IgG were added to the wells and incubated at room temperature for 2 hours with 400 rpm agitation. Upon incubation, wells were washed four times and 100 μL of detection antibody was added for 1 h at 400 rpm. After 4 washes, 100 μL per well of substrate solution were added and incubated for 15 min, upon when the reaction was stopped by pipetting 100 μL of stop solution to each well. Absorbance was read at 450 nm using a Versamax plate reader.

### Statistical Analysis

**Figure 1:**
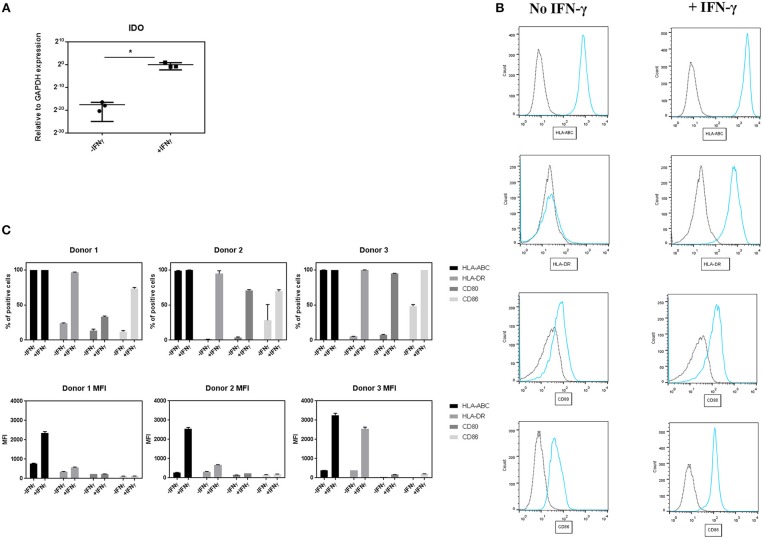
Effect of IFN-γ on the expression of immune regulatory and co-stimulatory markers on pMSCs upon IFN-γ stimulation. pMSCs treated with 50 ng/mL IFN-γ for 3 days or unprimed (-IFN-γ) pMSCs were analyzed for IDO gene expression by qRT-PCR, and stained for HLA-ABC, HLA-DR, CD80, and CD86 expression and analyzed by FACS. **(A)** Expression of gene encoding for IDO relative to GAPDH. *N* = 3 pMSCs donors, single replicates. **(B)** Representative flow cytometry histograms of HLA-DR, HLA-ABC, CD80, and CD86 expression (blue line) on pMSCs based on the unstained control (dot line) with and without the addition of IFN-γ. **(C)** Percentage of positive cells and Mean Fluorescence Intensity (MFI) for HLA-ABC, HLA-DR, CD80, and CD86 markers. *N* = 3 different pMSC donors in triplicates. Results are shown as means ± SD. *p < 0.05.

Data is expressed as mean ± Standard Deviation (SD), *n* = 3 (experimental replicates) in duplicates or triplicates unless otherwise stated, where *p* < 0.05 values were considered as statistically significant. For all B and T cell proliferation analysis, *N* = 3 PBMC/B cell donors and a minimum of *N* = 1 pMSC donors were used. Statistical analysis was performed by the software IBM SPSS Version 24 using a linear mixed model with Bonferroni post-correction test for all figures, except for Figure 1A for which a Mann-Whitney test was used. *P*-values are represented as ^***^p < 0.001, ^**^p < 0.01, ^*^p < 0.05.

## Results

### IFN-γ Pre-treatment Upregulates the Expression of IDO and Immune Related Markers on pMSCs

To study the effect of IFN-γ on the expression of immunomodulatory and co-stimulatory molecules on pMSCs, 50 ng/mL of IFN-γ was added to undifferentiated pMSCs for 3 days. After that time, the expression of IDO was quantified by qRT-PCR, and CD80, CD86, HLA-ABC, and HLA-DR surface levels were measured by FACS (Figure 1). Stimulation of pMSCs with IFN-γ significantly upregulated the gene expression levels of IDO (Figure 1A). Unprimed pMSC of all donors were highly positive for HLA-ABC, whereas for the rest of markers (CD80, CD86, and HLA-DR levels) unprimed cells expressed <50% of positive cells. Upon IFN-γ stimulation the percentage of positive cells of CD80, CD86, and HLA-DR was increased for all donors. We also found an increase in the expression levels per cell, expressed as the Mean Fluorescence Intensity (MFI) of HLA-ABC and HLA-DR for all donors (Figures 1B,C).

These results imply that IFN-γ pre-conditioning increases the expression of the immunomodulatory related enzyme IDO, but also the levels of HLA and co-stimulatory molecules involved in the activation of B and T cells on pMSCs.

### IFN-γ Pre-treatment on pMSCs Does Not Affect Their Immunomodulatory Properties Upon T Cells

**Figure 2:**
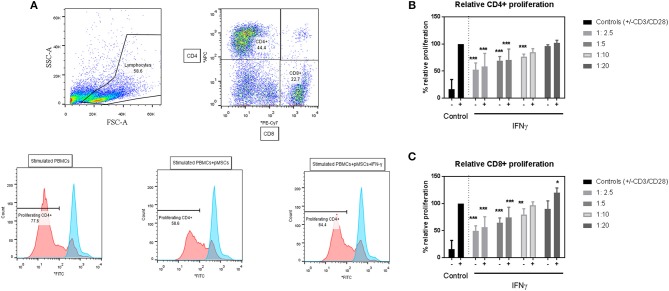
pMSCs are immunomodulatory toward T cells in a dose-dependent manner. CD3/CD28 stimulated PBMCs were co-cultured with IFN-γ primed (+IFN-γ) or unprimed (-IFN-γ) pMSCs at 1:2.5, 1:5, 1:10, and 1:20 pMSCs:PBMCs ratios. Flow cytometric analysis was performed after 5 days of co-culture, and CD4^+^ and CD8^+^ proliferating T cells were detected by CFSE. **(A)** Representative FACS plots and histograms showing the gating strategy for stimulated CD4^+^ and CD8^+^ T cells alone or in co-culture with unprimed or IFN-γ primed pMSCs. **(B)** CD4^+^ and **(C)** CD8^+^ T cell proliferation in co-culture with unprimed or IFN-γ primed pMSCs. Results are expressed as the relative proliferation measured as the 1/Mean Fluorescence Intensity (MFI) of CFSE normalized to the (+CD3/CD28) stimulated control. *N* = 3 different pMSC donors with *N* = 3 different PBMC donors in triplicates. Results are represented as means ± SD. *p < 0.05, **p < 0.01, ***p < 0.001.

To assess the immunomodulatory abilities of pMSCs toward T cells under inflammatory conditions, IFN-γ primed or unprimed pMSCs were co-cultured with stimulated PBMCs (+CD3/CD28 antibodies) at 1:2.5, 1:5, 1:10, and 1:20 pMSC:PBMC for 5 days (Figure 2). The proliferation of CD4^+^ and CD8^+^ T cells was tracked using CFSE. The reduction in T cell proliferation for both CD4^+^ (Figure 2B) and CD8^+^ (Figure 2C) T cell subsets induced by pMSCs was dose dependent for IFN-γ primed or unprimed pMSCs co-cultures. However, there was not a significant difference between the IFN-γ primed or unprimed conditions for any of the doses tested. Hence, we concluded that pMSCs are able to exert their immunomodulatory abilities toward T cells regardless of the addition of IFN-γ.

### IFN-γ Primed pMSCs Significantly Reduce the Proliferation Rates of B Cells

To examine whether inflammatory conditions could influence the anti-proliferative capacities of pMSCs on B cells, we co-cultured IFN-γ primed pMSCs with stimulated B cells for 7 days. We hypothesized that IFN-γ primed pMSCs would have similar immunomodulatory capacities on B cells compared to aMSCs (Luk et al., 2017).

**Figure 3:**
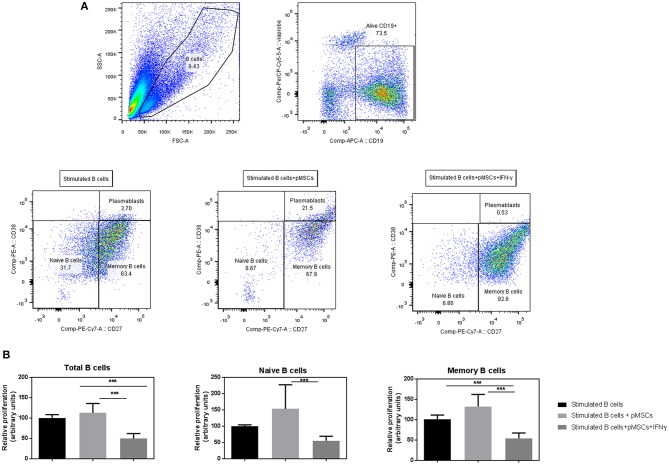
IFN-γ primed pMSCs significantly reduce the proliferation rates on naïve, memory and total numbers of B cells compared to unprimed cells. Stimulated (+anti-CD40, anti-IgM, and IL-2) B cells were co-cultured with either IFN-γ primed or unprimed pMSCs. B cells were retrieved and analyzed by flow cytometry after 7 days. **(A)** FACS gating strategy of viable CD19^+^ B cells that were classified according to the expression of CD27 into memory (CD27^+^), naïve (CD27^−^) B cells, or plasmablasts (CD27^high^ CD38^high^). **(B)** Relative proliferation of B cells when co-cultured with IFN-γ primed or unprimed pMSCs. Results are shown as the 1/MFI (CFSE) of CD19^+^ viable cells (total B cells), CD27^+^ cells (memory), and CD27^−^ cells (naïve) relative to the B cell stimulated condition. *N* = 3 B cell donors co-cultured with 1 pMSC donor in duplicates or triplicates. Figures show means ± SD. ***p < 0.001.

B cells were stimulated with anti-CD40, anti-IgM, and IL-2 to mimic T cell activation in the absence of T cells. Viable B cells (Viaprobe^−^ CD19^+^) were analyzed by FACS and classified into CD27^+^ (memory) and CD27^−^ (naïve) B cells or plasmablasts (CD27^high^ CD38^high^) (Figure 3A).

Co-culture of unprimed pMSCs with B cells did not reduce proliferation rates of total B cells, naïve or memory B cells, but a tendency toward an increased proliferation was observed compared to stimulated B cells on their own. However, IFN-γ pre-conditioning of pMSCs decreased the proliferation rates of naïve, memory and total B cells in co-cultures compared to stimulated B cells. Moreover, IFN- γ primed pMSCs significantly decreased the proliferation rates of all B cell subsets when compared to unprimed pMSCs (Figure 3B).

### IFN-γ Primed pMSCs Abrogate Plasmablast Differentiation and IgG Production

After 7 days of co-culture with IFN-γ primed or unprimed pMSCs, we determined the frequencies of CD19^+^CD27^high^CD38^high^ plasmablast cells by FACS (Figure 3A). In order to characterize B cell functionality, we quantified the amount of antibody released by B cells by measuring the IgG production in the supernatants of the co-cultures by ELISA.

**Figure 4:**
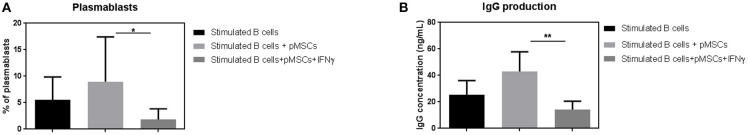
IFN-γ primed pMSCs but not unprimed significantly decrease plasmablast differentiation and IgG production. **(A)** Plasmablast frequencies were measured upon 7 days of co-culture of stimulated B cells with IFN-γ primed or unprimed pMSCs. Results are expressed as the % of viable CD19^+^ CD27^high^ CD38^high^ cells. **(B)** IgG concentration (ng/mL) was detected in the supernatants of stimulated B cells and IFN-γ primed or unprimed pMSCs by ELISA. *N* = 3 different B cell donors co-cultured with 1 pMSC donor Results are shown as means ± SD. *p < 0.05; **p < 0.01.

Unprimed pMSCs did not significantly reduce the number of plasmablasts when co-cultured together with stimulated B cells (Figure 4A). However, there was a statistically significant reduction in the percentage of plasmablasts detected when co-cultured with IFN-γ primed pMSCs (8.9% of plasmablasts vs. 1.8%, respectively). A significant reduction in the amount of IgG present in the supernatants was also found when B cells were co-cultured with IFN-γ primed pMSCs compared to unprimed pMSCs, from 43 to 14 ng/mL (Figure 4B).

## Discussion

The immunomodulatory abilities of MSCs upon the adaptive immune response have been previously investigated in a number of studies (Bartholomew et al., 2002; Su et al., 2013; Franquesa et al., 2015; Luk et al., 2017). Moreover, MSCs immunomodulatory properties upon T and B cells have been shown to be highly influenced by the local inflammatory microenvironment (Krampera et al., 2006). These studies have reported some promising alternatives to tailor the immunosuppressive abilities of MSCs, by subjecting them to certain pro-inflammatory cytokines such as IFN-γ (Tipnis et al., 2010; Luk et al., 2017). However, they were performed using MSCs derived from diverse sources of aMSC donors, which can lead to high variability and unpredictable results due to a large age and origin variation among donors (Bruna et al., 2016), as well as reduced immunomodulatory abilities associated to age (Wu et al., 2014). We have previously established that the use of a potent source of pMSCs with enhanced multilineage differentiation and expansion abilities, as well as reduced senescence, could make them attractive candidates for regenerative medicine (Knuth et al., 2018). Moreover, in the same study pMSCs were shown to reduce T cell proliferation in an allogeneic *in vitro* co-culture model. Here, we aimed to further investigate the immunomodulatory abilities of pMSCs toward T and B cells under inflammatory conditions as a promising MSC source for regenerative medicine and allogeneic transplantation with more consistent differentiation abilities and increased expansion properties.

Inflammatory signals have been suggested to alter the immunomodulatory functionality of MSCs toward B cells (Krampera et al., 2006). Luk et al. (2017) previously showed that IFN-γ primed adipose derived aMSCs inhibited naïve (CD19^+^CD27^−^) and memory (CD19^+^CD27^+^) B cell proliferation. In this study we clarified the immunomodulatory abilities of pMSCs upon stimulated B cells. Our findings suggest that IFN-γ priming is crucial for pMSCs in order to exert their immunomodulatory functionality upon B cells. IFN-γ primed pMSCs but not unprimed pMSCs showed a significant reduction in B cell proliferation for all different B cell subsets.

Franquesa et al. (2015) previously reported that in the presence of adipose-derived MSCs, antibody producing plasmablast (CD19^+^CD27^high^CD38^high^) formation was inhibited. Hence, we hypothesized that pMSCs would also reduce plasmablast differentiation and that this effect would be enhanced by IFN-γ pre-priming. Our results showed that pMSCs reduced the numbers of plasmablasts only upon priming them with IFN-γ. We also showed that antibody production was significantly decreased when IFN-γ primed pMSCs were present and not in the presence of unprimed pMSCs. Since plasmablasts possess an antibody-producing functionality, these results suggest that in the case of an inflammatory microenvironment pMSCs significantly suppress humoral responses mediated by B cells. Moreover, since IFN-γ can be produced by T cells upon activation, these results suggest that the cross-talk between B and T cells might be of importance as a potential source of IFN-γ that can promote pMSC's immunosuppressive mechanisms upon B cells. In the situation of an allogeneic transplant, these properties of pMSCs might be advantageous to avoid allo-antibody formation against the allograft, one of the principal consequences of rejection and graft vs. host disease (GvHD) (Young et al., 2012). This knowledge might also be useful in the clinical context where an acute or chronic B cell mediated inflammation is present, such as autoimmune diseases or osteoarthritis.

MSCs are also well known to express major histocompatibility complex (MHC) class I molecules on their surface, but to have a minimal expression of MHC class II (Le Blanc et al., 2003) and co-stimulatory molecules such as CD80 and CD86 (Chamberlain et al., 2007). Addition of IFN-γ has been reported to enhance the expression of MHC class I and II (Majumdar et al., 2003), but it does not seem to increase the levels of CD80 and CD86 (Chinnadurai et al., 2014). Here, we show that unprimed pMSCs express high levels of MHC class I (HLA-ABC) and low levels of MHC class II (HLA-DR), CD80 and CD86. However, IFN-γ stimulation upregulated the levels of HLA-DR and both co-stimulatory molecules on pMSCs. This increase could mean that pMSCs would be more prone to act as antigen presenting cells and activate T cells under inflammatory conditions (Lim et al., 2012). Many studies have discussed the crucial role of IFN- γ on MSCs immunomodulatory abilities toward T cells (Krampera et al., 2006; Ryan et al., 2007). Since an increased immunosuppressive effect from MSCs upon T cells under inflammatory conditions has been linked to a higher IDO activity (Meisel et al., 2004), we examined the gene expression of IDO on pre-primed pMSCs. Our results showed that, in contrast to previous studies (Ryan et al., 2007; Wobma et al., 2018), IFN-γ priming did not affect pMSCs immunomodulatory abilities upon T cells. However, IFN-γ significantly upregulated the gene expression of IDO in all pMSCs donors. Therefore, it is possible that the upregulation of HLA-DR, CD80, and CD86 triggered by IFN-γ on pMSCs counteracted the IDO mediated enhancement on their immunomodulatory abilities upon T cells. The extent of this upregulation was observed to be donor dependent. This indicates that intrinsic variation among pMSCs donors might impact the immunomodulatory abilities of pMSCs toward T cells according to the degree of expression of co-stimulatory molecules in an inverse manner. Despite this, under inflammatory conditions, pMSCs were still able to suppress T cell proliferation in a similar trend than unprimed pMSCs. Previous studies have reported as well that the immunomodulatory potency of MSCs is highly related to the inflammatory milieu created by T cells (Kronsteiner et al., 2011). Hence, the cytokine profile of T cells might in turn affect the immunomodulatory effect of MSCs on immune cells.

Differences in the immunomodulatory abilities of IFN- γ primed pMSCs compared to other sources of aMSCs may depend on multifactorial pathways that have been described to play a role in the degree of immunomodulation on MSCs toward T and B cells. Luk et al. (2017) previously reported that upon IFN-γ addition, MSCs inhibited B cell proliferation as well as IgG production and regulatory B cells (Breg) formation through the tryptophan depleting activity of the enzyme IDO. Some studies have reported the involvement of other immunomodulatory mechanisms, such as Galectin-9 (Gal-9) which significantly reduced IgG titers in an *in vivo* murine model (Ungerer et al., 2014). The Cyclooxygenase 2 (COX-2) pathway has also been indicated to play a role in Breg suppression through IL-10 depletion (Hermankova et al., 2016). Signaling pathways involving the release of metalloproteinase-processed CC-chemokine ligand 2 (CCL-2) by MSCs controlled by the downregulation of olfactory 1/early B cell factor–associated zinc-finger protein (OAZ) have been proposed as a mechanism of MSC inhibition of IgG synthesis on B cells (Feng et al., 2014).

Moreover, it is also important to remark that different extents in the increase of co-stimulatory molecules on pMSCs surface, which seems to be donor-dependent as presented in this study, might entail as well differences in IFN- γ primed pMSCs immunomodulatory abilities. This is due to the fact that a higher upregulation of these co-stimulatory molecules, such as CD80 and CD86, in certain donors may negatively impact the immunosuppressive potential of IFN- γ primed pMSCs. Hence, this intrinsic donor variability on the immune profile of pMSCs needs to be taken into account when considering using pMSCs for immune therapies.

Together, we show that IFN-γ priming exerts an impact on the expression of immune markers and co-stimulatory molecules on a novel source of pediatric MSCs. In this context, pMSCs maintain their immunomodulatory abilities upon T cells, significantly suppress B cell proliferation, as well as plasmablast differentiation and antibody production. *In vivo*, this might mean that upon receiving certain inflammatory signals, such as IFN-γ produced by activated T cells, pMSCs safeguard their immunomodulatory status toward T cells, and gain anti-proliferative abilities upon B cells, avoiding an allo-antibody immune response. This knowledge is useful for the design of novel immunotherapies in several types of diseases where a chronic inflammation is present, as well as in the areas of regenerative medicine and solid organ transplantation by using a novel MSC source with enhanced differentiation abilities and an immune privileged condition.

## Data Availability

The datasets generated for this study are available on request to the corresponding author.

## Author Contributions

VP: conception and design, collection of data, data analysis and interpretation, and manuscript writing. MH: conception and design, data analysis and interpretation, and manuscript writing. RK: collection of data and final approval of manuscript. MF: conception and design, data analysis and interpretation, and final approval of manuscript. JW-B: collection of data and final approval of manuscript. EW: conception, manuscript writing, and final approval of manuscript. EF: conception and design, data analysis and interpretation, and manuscript writing. PB: conception, data analysis and interpretation, manuscript writing, and final approval of manuscript.

### Conflict of Interest Statement

The authors declare that the research was conducted in the absence of any commercial or financial relationships that could be construed as a potential conflict of interest. The reviewer GE declared a shared affiliation, with no collaboration, with several of the authors, VP and PB, to the handling editor at the time of review.
